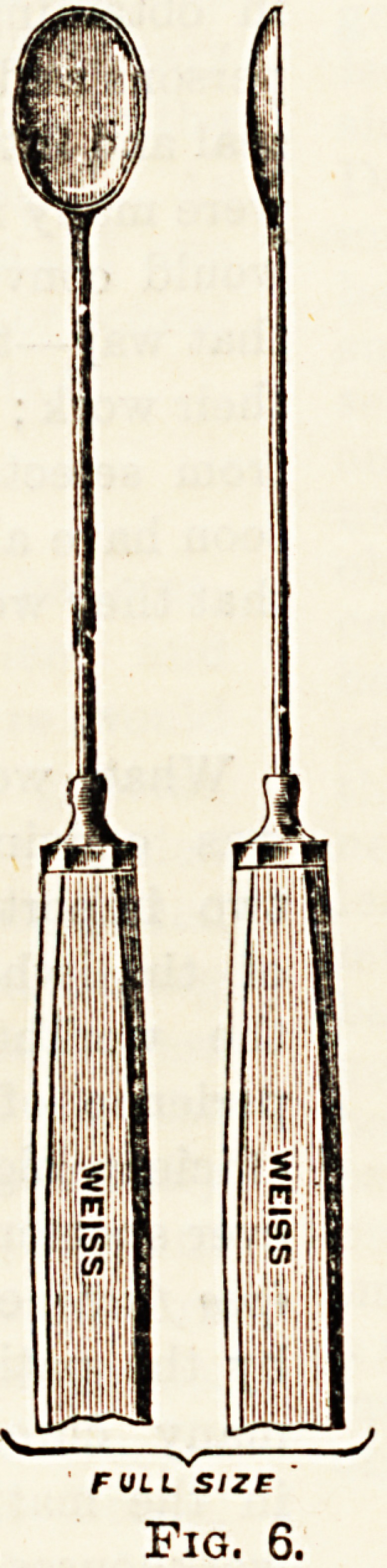# The Hospital. Nursing Section

**Published:** 1903-11-07

**Authors:** 


The Hospital.

Nursing Section.

Contributions for this Section of "The Hospital" should be addressed to the Editor, "The Hospital" Nursing Section, 28 & 29 Southampton Street, Strand, London, W.C.

No. 893.—Vol. XXXV. *SATURDAY, NOVEMBER* 7, 1903.

### Notes on News from the Nursing World.

### OUR CHRISTMAS DISTRIBUTION.

As some of our readers seem to be in doubt which articles are most likely to be acceptable to the matrons of hospitals and infirmaries, who will receive a parcel when we distribute the contributions forwarded to us, it may be useful to mention that warm under-garments of any kind are invariably keenly appreciated. It need not be feared that the supply of these will be in excess of the demand. On the contrary, the demand increases every year, and, with the constant opening of new isolation hospitals, will continue to become greater. We can only hope to be the instrument of helping a few of the many to whom the gift of a flannel shirt or petticoat means more than those who have always an abundance of warm clothing can imagine. Since last week we have received parcels from :— Miss Knight, 42 Anson Road, Tufnell Park, N. ; Miss N. C. Grist, 5 Lonsdale Terrace, Penrith ; Miss Colhoun, Monks' Hatch, Horsham ; and Nurse Besant, 4 York Street Chambers, London.

### THE STANDARD OF WORKHOUSE NURSING.

The fact that at the large and influential meeting which was held last week at St. Thomas's Hospital, under the auspices of the Hospitals Association, a resolution was unanimously passed disagreeing with the proposal of the Departmental Committee on Nursing to create minor training schools in small workhouses, and the recommendation that a probationer who has undergone one year's training in such schools should be considered and called a "qualified nurse," ought to seal the fate of a scheme which cannot be defended either in the interests of the nursing profession or in those of the public. It will be seen from our report of the discussion on the recommendations, that after Dr. Dixon Savill, whose experience as a medical superintendent of a workhouse infirmary entitles him to speak with authority, had shown that the effects of the recommendations, if carried out, would lower the standard of the whole nursing profession, Miss Gibson read an admirable paper in which she insisted upon the claims of the sick poor to the same careful and skilled nursing that is given to their more fortunate, but not more suffering, fellow-creatures. The matron of Birmingham Infirmary urged, with unanswerable force, that though the formation of a new grade of half-trained nurses might only at the beginning affect probationers in workhouse infirmaries, it must in the long run add to the number, already far too great, of unqualified persons having a so-called certificate and calling themselves "nurses." We necessarily hold over a portion of our report, but in the course of the discussion which followed the reading of Miss Gibson's paper, Miss Baker said that one of the minor training schools suggested in the Departmental report had already been started at the St. Luke's Workhouse Infirmary, where there were 80 infirm and chronic cases. It is, therefore, extremely important that the President of the Local Government Board should be pressed to lose no time in coming to a decision, and we hope that Mr. Long will be prepared not only to receive the deputation suggested at the recent meeting, but also to intimate that the recommendations denounced by all the heads of the nursing profession, as well as by many of the most eminent of medical authorities, will not be acted upon.

### VICEREGAL VISIT TO SIR PATRICK DUN'S HOSPITAL.

On Thursday last week the Lord Lieutenant of Ireland and the Countess of Dudley paid a visit to the interesting old hospital in Dublin founded by the late Sir Patrick Dun in 1800. Their Excellencies arrived at noon and were received by the chairman, governors, medical staff, and lady superintendent of the hospital. After having been given a short history of the place the Viceregal party visited the various wards. Lady Dudley spoke to each of the patients and gave them all flowers. The interest she takes in the sick poor is well known, and the patients much appreciated her visit and were gratified by the attention shown. Many of them came from the counties of Mayo, Sligo, or Donegal, and Her Excellency's visit to them in the wards of the "big hospital" will be a long-cherished remembrance. The operating theatre, built in 1896, *x*-ray rooms, and the new out-patients' department were all seen, but the nurses' home could not be visited as the covered way does not extend the whole distance and the day was wet. The wards all looked cheerful and bright. Lady Dudley expressed admiration for the scarlet jerseys worn by the children.

### THE WALTHAM TRAINING SCHOOL AND HOME NURSING.

In the report and description of the Waltham Training School for 1903 convincing proof of the still growing popularity of the institution is afforded. Thus, last year, the Waltham nurses were employed 13,664 days as against 12,026 the previous year, and 10,080 in the year ending June, 1901. The demand for the services of the student nurses, it is stated in the report, so far exceeds the supply that it is not unusual to have to refuse a hundred calls in a month. An equally satisfactory account is given by the treasurer with regard to the earnings, which reached the total of 17,326.11 dollars as against 14,247.45 the previous year. One of the distinctive characteristics of the Waltham School is of course the training given outside the hospital; and, in the face of adverse opinion from the nursing profession in the United States concerning this feature, the managers set forth their reasons for clinging to it "as the most valuable part of the nurse's training." In the first place, they affirm that the Waltham method in its outside training closely follows Fliedner's method at Kaiserswerth, where Florence Nightingale was trained, and also Susan Dimock's method, in which the first American nurse, Miss Linda Richards, was trained. The success achieved by the method, both in Waltham and in many other places where it has been adopted, in meeting the needs of the community is cited as a second reason. This success, it is pointed out, is shown not only in the increasing demand for the services of both students and graduates, but even more emphatically in the establishment of independent organisations for carrying on the different kinds of nursing work developed by the training school. The fact that the different universities about to undertake the education of nurses are all inclined to adopt the preparatory year and the training in home nursing which are of the essence of the Waltham plan, is quoted as a final proof of the excellence of the method in force.

### THE LATE SIR GEORGE GUNNING.

By the death of Sir George Gunning, at the age of 75, the nursing movement in Northamptonshire has lost a warm and generous friend. Sir George was one of the most strenuous supporters of the Northampton Town and County Nursing Institution, whose claims upon the public he was never tired of urging. He was foremost in pressing on the movement for the establishment of the Jubilee Nurses' Home, and it was a source of great satisfaction to him when the town and county united in order to carry it to a successful issue. He rendered active assistance in the arrangements for its opening by Princess Christian, whose health on the occasion of her visit to Northampton he happily proposed.

### THE CLUB QUESTION IN DUBLIN.

The need of a nurses' club in Dublin is urged by a Dublin nurse, who states that the institution which formerly existed at St. Stephen's Green was greatly appreciated, particularly by private nurses. If so, however, why was it not supported more adequately? We can quite understand that there are many private nurses in the Irish capital who are willing to pay for the privilege of being in touch with the doctors through the telephone, of reading the papers, and of enjoying other conveniences. At the same time, a club must be run on business principles; and if an undertaking, with much to commend it, which was started for the use of nurses did not attract a sufficient number to make it pay, we do not wonder that there is some hesitation in repeating the experiment.

### NURSES AS STEWARDESSES.

It seems that the number of nurses with more or less training who desire to obtain appointments as stewardesses is very large. We are informed by the Secretary of the Royal Mail Steam Packet Company, who advertised for nurses as stewardesses, that so far the applicants exceed 400 in number. The idea in advertising, he states, was to give the preference "to those nurses who had been through an all-round hospital training, rather than maternity nurses or others whose experience was more or less limited." With over 400 candidates for posts, the company naturally came to the conclusion that the supply is greater than the demand.

### A NEW MATRON AT KING'S LYNN HOSPITAL.

The list of appointments announced in our columns this week includes that of a new matron of the King's Lynn and West Norfolk Hospital. Miss Helen Swain, who, it may be hoped, will find it possible to restore to this institution some of the prestige which it has lately seemed to have lost, so far as the nursing staff is concerned, was trained at the Royal Berkshire Hospital, has been sister and night superintendent at Worcester General Infirmary, and has done nursing in South Africa as a member of the Army Nursing Service Reserve. When she left Worcester she received a presentation of Worcester China, to which the matron, sisters, nurses and doctors subscribed. We hope that in her new post of greater responsibility she will achieve success.

### OPENING OF A NEW HOME AT GRANTHAM.

The Mayor of Grantham last week opened the nurses' home which has been erected in the town as a memorial to Queen Victoria. The building is situated in Castle Gate, is in the Gothic style of architecture, and is convenient and commodious. On the ground-floor there are a small room for the reception of visitors, the nurses' sitting-room, and the board-room; on the first-floor four bedrooms and a bath-room; and on the second two attics. It was announced at the opening ceremony that the cost of the undertaking, about £1,000, had been defrayed by residents of Grantham and the neighbourhood. The architect gave his advice gratuitously, the builder used more expensive material than was contracted for, and the Mayor, having first contributed £200 to the home, has since devoted his official salary of £50 to the furnishing fund of the institution.

### THE NURSING STAFF AT CAMBERWELL INFIRMARY.

The Infirmary Committee of the Camberwell Guardians have received a report from the medical superintendent who states that, having considered the question of the nursing staff for the infirmary, when complete, for 800 beds, and remembering the class of case, medical and surgical, that will have to be treated, together with the requirements of a modern nursing staff, he could make no suggestion with a view to a reduction of the number of officers needed. In these circumstances we think that the Guardians have wisely resolved to again urge the Local Government Board to sanction the scheme as originally submitted for the staffing of the infirmary, and we hope that the sanction of the Board may be obtained.

### OVERCROWDING IN A NURSES' HOME.

It is perhaps inevitable that there should be overcrowding in a nurses' home attached to a workhouse which has unexpectedly proved to be insufficient for the requirements. This, it appears, is the case at Wolverhampton, and the Guardians are now face to face with the problem of having to provide further accommodation for the nursing staff. A home loses all its advantages if the nurses do not have a separate bedroom, or if the sitting-rooms are inconveniently full during the day. The unfortunate experience of the Wolverhampton Guardians should serve as a warning to other bodies to be careful to take into the most careful consideration the possibilities of the future.

### THE MODIFIED OATH.

The graduating nurses of the Bucks Memorial Hospital, Dunkirk, New York State, are required to take the following "modified oath":—"I solemnly promise and swear that in the practice of my profession I will always be loyal to the patients entrusted to my care and to the physicians under whom I shall serve; that I will not make use of nor recommend any quack or secret nostrum; that I will be just and generous to members of my profession, aiding them whenever they shall need aid, and I can do so without detriment to myself or patient; that I will lead my life, and practise my profession in uprightness and honour, and not lend my aid to any criminal or illegal practice whatever; that into whatever house I shall enter, it shall be for the good of the sick to the utmost of my power; that whatever I shall hear or see of the lives of men or women, whether they be my patients or members of their households, that I will keep inviolably secret; and that I will continue to observe and to study and will strive in every way for the improvement and advancement of my profession, not regarding it as a means of livelihood only, but as an honourable and sacred calling."

### DISTRICT NURSING AT BARNSTAPLE.

It was announced at the fifth annual meeting of the Barnstaple District Nursing Association that the visits paid in the year had been 3,182, or 44 more than in the preceding year. Of the 201 patients visited by the nurse 158 were reported convalescent, 23 were transferred to the hospital or workhouse, and 20 died. Including £11 14s. 9d. brought forward, the year's income amounted to £111 18s. 10d. and the expenses were £109 8s. 3d. The association has now £106 on deposit. This is a useful nest-egg, but as the accrued interest on the deposit had to be drawn this year, there is evidently room for more subscribers to the excellent work being done in the district.

### THE ANNUAL SALE AT PECKHAM.

The third annual meeting of the Peckham Nursing Association was held last week. The financial statement, which was submitted by Mr. Charles Ward, the assistant treasurer, showed that the income was £160 18s. 1d., being £25 in excess of the previous year. The expenditure amounted to £107 14s. 2d., and it was therefore decided that an addition of £40 should be placed to the proposed Nurses' Home Fund account, which now stands at £100. The receipts included £49 5s., the proceeds of a sale arranged by the ladies of the association, but as the sale is an annual event, we suppose that it may be regarded as an ordinary source of income. The increase in the number of patients, seven of whom suffered from cancer, has already suggested that the necessity of appointing a second nurse will soon arise, and the importance of augmenting the home fund is therefore obvious.

### VILLAGE NURSES IN CUMBERLAND.

At the sixth annual meeting of the Cumberland Nursing Association it was stated that eight new associations had been formed during the year. Three more were waiting for a nurse to be trained for them before they could start. At present there are eight candidates undergoing a course of training at Plaistow, half required for new posts and half to fill vacancies in connection with existing associations. The present association has now 33 nurses at work in districts affiliated to the County Association, six Queen's nurses and 27 village nurses, all of whom are certified midwives with at least nine months' midwifery and general training. Some have also had a year's experience in a hospital or workhouse. The report states that out of the £1,300 realized by the bazaar in September of last year £300 is being used to train nurses, and £1,000 has been invested. The County Council has given two scholarships of £50 each for training two "village nurses," and the Carlisle Board of Guardians £15 for distribution in districts employing village nurses in the Carlisle Union. Compliments were paid at the meeting to the Countess of Lonsdale, the president, who was unavoidably absent.

### DISTRICT NURSING AT CHEADLE.

At the thirteenth annual meeting of the Cheadle and Gatley District Nursing Association, the hon. treasurer, in submitting her report, stated that without special contributions, such as those of the Cheadle Dramatic Association for £5, the Loyal Conciliation Lodge of the I.O.U. Foresters for £2 1s. 10d., and the proceeds of a jumble sale, which realised £29, there would have been a balance on the wrong side, instead of a balance of £13 in hand. She was sorry to say that there had been a decrease in the subscriptions. This, in spite of special help, is always a bad sign, and indicates the need of fresh efforts to maintain, or increase, the regular income of an organisation. As the district nurse paid 2,388 visits to 177 cases, and her services seem to be greatly appreciated by the sick poor, there ought to be no slackness in finding sufficient money for her salary and expenses.

### A SCHOOL FOR MASSAGE IN LIVERPOOL.

As we have frequently been asked whether there is a school for massage in Liverpool, it may be interesting to some of our readers to know that Miss Hunter, who was until lately assistant matron at the Royal Berkshire Hospital, and is a member of the Incorporated Society of Trained Masseuses, will early in December endeavour to supply the want. In addition to instruction in massage and elementary anatomy and physiology, candidates will be prepared under her auspices for the examination of the Society of Trained Masseuses.

### SHORT ITEMS.

The s.s. *Saxon* arrived at Southampton on Saturday from South Africa, having on board Sisters G. Romer and A. G. Storie, of the Army Nursing Service Reserve. Their time has expired.—Miss M. H. McLeish has resigned her appointment as Sister of Queen Alexandra's Imperial Military Nursing Service.

### Lectures on Ophthalmic Nursing.

By A. S. Cobbledick, M.D., B.S.Lond., Senior Clinical Assistant and late House-Surgeon and Registrar to the Royal Eye Hospital.

### LECTURE XXII.—TREATMENT OF CATARACT.

(*Continued*)

1. Cataracts occurring in early life before the formation of a nucleus are treated by the method of discission. As was mentioned in the last lecture, this consists in opening the lens capsule and breaking up the lens substance with a needle.

For this operation, infants and young children should be placed under a general anæsthetic, the best of which is chloroform, and the pupil should have been previously dilated by the use of atropine. The only instruments required are an eye speculum, a pair of fixation forceps, and an arrow-pointed needle, with or without a stop on it (figs. 1 and 2).

The usual routine of sterilization of instruments and washing out of the conjunctival sac must be followed. When the eye speculum is applied and the eyeball fixed with forceps, the needle is passed obliquely through the cornea a few millimetres from the outer margin. By moving the point of the needle from above down, the capsule is opened; a series of rotatory movements are next made so as to thoroughly break up the lens substance.

Figs. 1 and 2. Fig. 3. Fig. 4. Fig. 5. Fig. 6.

The needle is then carefully withdrawn, so that the arrow point follows the same path as on entry, otherwise a small crucial incision results through which some leakage of aqueous may occur.

When the operation is completed another drop of atropine is instilled and the eye kept covered until the following day.

As a rule there is not much reaction, but at times considerable deep injection results: when this is the case, iced boracic flaps frequently applied to the eye give most relief.

In a few hours the absorption of the aqueous by the lens produces marked opacity, and individual pieces of broken-up lens may be seen.

Atropine gr. i. or ii. ad. ʒ i., must be continued night and morning, in order to keep the free border of the iris from coming in contact with the lens. During the subsequent three or four weeks, considerable absorption of the lens substance takes place, and some idea of the amount of unabsorbed debris, which is likely to remain, is obtained. Some cases require a second needling. In the course of two or three months' time it is usually found necessary to make a small cut through the remaining debris and capsule, in order to get the best vision; this operation is termed *capsulotomy*.

A capsulotomy needle has a long cutting edge on one side only (fig. 3). It is advisable in performing this operation to fix the capsule with a needle, held in the left hand, before making an incision with the capsulotomy knife; otherwise, if the capsule and *débris* are tough, too much traction is made on the attachment of the capsule to the ciliary body, and inflammatory trouble may follow.

Traumatic cataracts in adults may or may not require to be needled; if the lens substance escapes into the anterior chamber as the result of the accident, or of a needling, some absorption takes place, but frequently the tension rises considerably, and evacuation through an incision in the cornea must be resorted to.

2. *Extraction of Cataracts.*—As was pointed out in the last lecture, one of three different methods may be followed. As the operation of iridectomy has been previously described, the extraction only will be dealt with here. It is important to recollect that, before any steps towards extraction are taken, the presence of chronic conjunctivitis, or dacryocystitis, must be excluded. Inattention to these points has been the cause of spoiling what might have been a useful eye. Two other important points are the presence of cough—not infrequent in old people—and the condition of the urine; the latter should be examined in every case.

Assuming that a preliminary iridectomy has been performed, the instruments now required are a Graefe cataract knife, an eye speculum, a pair of fixation forceps, a cystitome (fig. 4) wherewith to open the lens capsule, a small, smooth curette, a tortoise-shell spoon (fig. 5). The curette and the cystitome may be conveniently fixed on the one handle (fig. 4). A larger spoon should also be ready in case of emergency (fig. 6).

After the speculum is inserted and the eyeball firmly fixed with forceps, the section is made in the same manner as described in performing iridectomy for glaucoma, but the flap must be larger. One of the most common mistakes is to make too small a section, so that the cataract cannot be extracted unless the incision is enlarged.

When the section is completed the cystitome is introduced into the anterior chamber and the point lightly drawn across the anterior capsule and from below upwards in withdrawing it, thus the lens is free to escape into the anterior chamber. Light pressure is exerted over the lower part of the cornea with the small smooth spoon so as to slightly separate the edges of the section and also to tilt the upper extremity of the cataract forwards and engage it in the section. Directly the cataract has escaped the speculum should be released. If the cataract is not quite matured some cortical substance is usually left behind; some of this can be expressed by gently moving the lower lid over the cornea and exerting a slight pressure upwards.

If the iris is caught in the angles of the incision it should be carefully replaced with a curette. A pad and bandage must next be applied to both eyes and left untouched for three days; the pad may then be removed from the sound eye and the condition of the eye operated upon inspected.

### Workhouse Nursing.

### THE HOSPITALS ASSOCIATION AND THE REPORT OF THE DEPARTMENTAL COMMITTEE.

In the Governors' Hall of St. Thomas's Hospital on Thursday, October 29th, a discussion took place under the auspices of the Hospitals Association on the Report of the Departmental Committee on Nursing appointed by the President of the Local Government Board. In the absence of Sir Henry Burdett, who was unable to be present, Mr. Bryant, F.R.C.S., took the chair. There was a large attendance, including Mr. J. G. Wainwright, Treasurer of St. Thomas'; Mr. H. Bonham Carter, Secretary of the Nightingale Fund; Lady Belhaven, Mr. Theodore Acland, Miss Gibson, Miss Gill, and many Poor-law Guardians, matrons and other representatives of the nursing profession. The discussion was very animated, and lasted nearly three hours.

In opening the proceedings, the Chairman said that no doubt they had all read the report they had met to consider, or they would hardly be in a position to discuss the subject with which it dealt—the nursing of the sick poor in workhouses, and the proposals put forward for its improvement.

### Magnitude of the Question.

Dr. T. Dixon Savill commenced the discussion. He said he would like to remind them of the magnitude of the question with which they were dealing. There were in the metropolis from 13,000 to 14,000 beds provided for the sick poor—a larger number than those in all the general and private hospitals; while it was estimated that in England and Wales the number amounted to 75,000 directly or indirectly affected. The question to which their attention was directed was how best these people were to be nursed, and how best the necessary nurses might be obtained. Probably all there were aware that the reason the report was called for was because of the deficiency in the supply of nurses in England and Wales, and this deficiency the report admitted—naturally with difficulty and with many qualifications—but it was admitted, and that was the first and fundamental point. It was only when they came to the question of a remedy for this deficiency in the supply of nurses that trouble arose. The next part of the report dealt with the training of nurses in workhouses, and suggested that there should be major and minor training schools in workhouse infirmaries. The major schools had been in work for a good many years, but the proposal to institute minor training schools was one of the two points to which he specially wanted to direct their attention. It was suggested that the smaller workhouses throughout the kingdom, with less than 60 beds, should be constituted minor training schools, provided they had a resident superintendent nurse and a visiting—not a resident—doctor. It was further suggested that probationers should be regarded as qualified nurses after one year's training at one of these minor schools. After that the Report went on to deal with a variety of matters of a technical kind, and to make some valuable suggestions to improve the conditions of service in workhouses. It also dealt with the questions of the joint appointment of matron and trained nurse, midwifery qualifications, and grants in aid of salaries. In brief, that was the sum and substance of the report.

### Constitution of the Committee.

On the whole subject there were two points to which he particularly wished to draw attention. Firstly, the committee was not one to command confidence. It consisted of two medical inspectors of workhouses, one former inspector, and the Chairman, the Parliamentary Under-Secretary—all Local Government Board officials, without a single outside nominee; and since the workhouses and infirmaries were under the care of that department it was like asking the father of a family what he thought of the conduct of his girls. There was not a single nursing expert on the committee, although the main question they had to consider was how to provide nurses for workhouses. Had they sought the co-operation of the great training schools, either in the metropolis or the provinces, they would have inspired more confidence. There was not even one woman among their number, and so these worthy gentlemen had had to do their best and blunder through as men generally did in women's affairs, with the consequence that they had got hold of the wrong end of the stick, and their report did not command the confidence of any impartial observer.

### The Minor Training Schools.

Secondly, he would mention the objections he and many others had to the minor training-school scheme. These small workhouses were deficient in the material means for technical education, the field for patients was limited, and medical and surgical treatment, if it went on at all, was necessarily not up-to-date. Then 12 months was a ridiculously inadequate time for the probationers to serve, and to call them qualified nurses at the end of that time was a complete misnomer. But the greatest blot of all was that it was not possible to infuse into the nurses the proper character, tone, and training which were the basis of proper nursing. Half the battle in obtaining good nurses was in the selection of suitable persons and the moral education given them. They required zeal and interest in their work, *morale*, and discipline. There were many intelligent women whom no amount of experience would convert into good nurses, because they were not built that way—they did not take the necessary view and tone of their work; and since there was nothing to prevent a matron from selecting pauper inmates as probationers they would soon have all the old evils flourishing, with this in addition, that they would have to pay for them.

### The Effect of the Proposals.

What would be the effect of these proposals? He was convinced that they were against the interests of two important classes, and so opposed to the interests of the whole community. First, as to their effect on the workhouses and guardians. Those who had experience of Poor Law working knew that the effect of efficient, high-toned nursing was most economical. Whatever shortened the stay of a patient in the workhouse was, *ipso facto*, economical. It was a matter of experience that by the patient and painstaking observation of the nurses many cures in troublesome cases were effected; while in the matter of malingering—always a great difficulty in workhouses—it was often only by the observation of a zealous nurse that the doctor was able to detect the imposture. In fact, he had no means but the day-to-day observation of a trained and skilful nurse. He commended this aspect of the case to the Guardians generally, and asked them to take an Imperial view of the whole question—not a narrow, parochial one—and he felt sure that a little co-operation among the various Boards of Guardians would go far to solve the whole question. Secondly, as to the effects of these proposals on the nursing profession. Here they would agree that the nurses must suffer. It was an insult to probationers to call them qualified nurses at the end of one year's training; they knew they were not qualified, and it would do the whole profession injury by lowering the standard. If these probationers were qualified by one year's training, then nurses generally were wasting two years of their life. Let them ask the doctors how they would like the medical standard lowered, and people to be

WORKHOUSE NURSING—*Continued.*

called doctors after 18 months' education instead of five years, on the ground that the poor needed medicine. It is not a question of class interest, but of the sufficient nursing of the indoor sick poor, and the suggestion was made that they should manufacture a cheap article for workhouse consumption. He had in his hand a paper by Dr. Downes, medical inspector of the Local Government Board and one of the Committee, from which he would like to read a passage which bore this out.

"The point [of providing for the sick poor] was very clearly hit by the late Lord Kimberley in his examination of a witness before the Select Committee of the House of Lords in 1890. The witness had complained that it was hard that the hospital-trained nurse 'should have to compete in the open market with so many amateurs.' 'But,' said Lord Kimberley, 'I have in my mind a vast number of poor people in the country who have to be nursed in their own homes; do you think it possible that the trained nurses who would be registered in such an association as yours could be employed in those homes? How are they to be paid? Would it be possible that any nurse who had gone through a training, which I admit is most valuable, in a hospital, could take the very small insignificant amounts which a poor man could afford to pay in his own cottage?'"

The reply to this was to point to the District Nursing Associations and to nurses all over the country who took inadequate remuneration, but who devoted their lives to ministering charity, with nothing but a lofty ideal to guide them. While referring to Dr. Downes's paper, he would like to express agreement with him as to the value of infirmary training in certain classes of cases—in chronic cases, in the treatment of bed-sores, and in the care of the aged. As Dr. Downes wrote, "he would rather be nursed in old age by a competent nurse from a well-organised minor school than by one from the smartest hospital for accidents."

### Lowering the Standard of Nursing.

Then as to the effect of these proposals on the community as a whole. In addition to the inefficient and therefore expensive nursing of the sick poor there would be the lowering of the standard of nursing in general, because there would be nothing to prevent these so-called nurses going into the open market. The report had evoked widespread disapproval among Boards of Guardians, among masters and matrons of workhouses, and in the press generally; and that disapproval was expressed in the memorial early this year, more extensively and influentially signed than any ever sent to a Government department before. But all this was of no use unless they could turn the Local Government Board from its pernicious proposals. What would Florence Nightingale say to all this? He thought she would say to the Local Government Board, Why did you not ask us? You would not have found us wanting. Why did you not consult some of the chiefs of the metropolitan training schools? To the Guardians she would say, Rise to the ideal that general efficiency means general economy. To the nurses she would say, Look to it that the ideals of your calling are not degraded. And to the public in general she would say, See that Great Britain, which has always been in the van of humanitarian principles, does not degenerate.

### Paper by Miss Gibson.

Miss Gibson, matron of Birmingham Infirmary, then read a paper, in the course of which she said:—

I believe that most people—certainly most experts—agree with most of the things I am going to say, but lack the occasion of saying them or dislike speaking in public too much to come forward and state their views. So I must beg for your forbearance while once again I endeavour to set forth the claims of the sick poor to the same careful and skilled nursing which is given to their more fortunate, but not more suffering, fellow-creatures. And at the moment this question is not only a question of guarding the poor, but also one of guarding the nurses themselves. The whole nursing profession is, or ought to be, deeply interested and involved in a suggestion which, though at the beginning it only affects probationers in workhouses, must in the long run lower the standard of teaching and add to the number—already far too great—of unqualified persons having a so-called certificate and calling themselves "Nurses." Skilled nursing is not, and ought not to be, a luxury for the rich; it is a necessity for all who are sick.

### The Parting of the Ways.

We have now come to the parting of the ways, and have, once and for all, to choose whether we take the right turning or the wrong. It also seems, alas! very likely that we may take the wrong. The suggestion of the Local Government Board Departmental Committee to form a new grade of nurse, to be called a "qualified nurse," seems to me dangerous and retrograde in the extreme. It is absolutely impossible, in the first place, that sufficient knowledge can be gained in one year in a small workhouse to make any woman a safe person to be left without supervision. No one can see the necessary variety of cases, or be called upon to deal with emergencies sufficient to give nerve and wisdom, in so short a time and with so small a variety of illnesses. I should like here to say that it is not sufficient to alter the name "qualified" nurse to do away with this danger. The name does not matter one bit, if the class remains. It cannot be for the good of the sick, or for the good of the nurse herself, that so low a standard of knowledge should be accepted, and it is a gross injustice to the sick poor. In the paper read last spring at Malvern, Dr. Downes stated, as a plea for the necessity for a shorter and more incomplete training: first, that "training can never be complete"—which seems a truism: we only plead for sufficient and adequate, not for complete training; and second, that "training must tend to become specialised"; and he seems to suggest that it will be sufficient to train nurses to nurse old age. This seems to be a somewhat obvious fallacy. Surely special knowledge must be preceded by general knowledge if it is to be of any value. I have always imagined that a specialist first underwent his general medical course, took his degrees, and so on, and then became a specialist. This I should also consider necessary for a nurse. She may, as Dr. Downes suggests, learn to nurse and devote herself to nursing old people in a minor training school, but she must also learn to nurse sick babies and surgical cases, and to attend operations—in fact, go through the ordinary general training which all must have who are to be called nurses at all. It must be remembered that a pneumonia, or an enteric, an amputation, or a hæmorrhage case may occur in any workhouse, however small, and however old the patients may as a rule be, and a nurse qualified in a minor training school, who has learned most skilfully to nurse only old people, in such a case is not only useless but positively dangerous.

### From the Public Point of View.

Then in regard to the public. I have no doubt whatever that the opening up of these minor schools will add very considerably to the already considerable number of only partly experienced persons, who join private nursing and other institutions, and who go out to nurse the sick in private hospitals and houses. For I read in the report of the Departmental Committee that at the end of a year's training in a minor training school a nurse may receive, first, "a certificate of good conduct and proficiency from the superintendent nurse, countersigned by the chairman of the Board of Guardians," and, second, a "certificate signed by the medical officer attached to the infirmary, stating that he considers the probationer qualified to undertake the ordinary duties of a nurse." I further notice that the medical officer must engage to devote some of his time to "instructing probationers by lecture or otherwise." This seems far too vague and illusory, and may mean something or nothing. But the point which I desire to emphasise is this: who is to discriminate between this certificate—given after one year in a small workhouse—and the certificate given after a training, probably much longer and certainly much more efficient, in a hospital or large infirmary? Certainly not the public.

I have heard it said—and I allow that there is some justification for it—that if the public desires a trained nurse, it ought to see that it gets one; and I have even heard it argued that, as it would be your own fault if you bought one thing when you needed another, so it was also your own fault if, needing a trained nurse, you took a half-trained one. This, however, is really another fallacy, for people are seldom able deliberately to set forth to make inquiries about a nurse. She is usually wanted at once, and most people are only too glad to send to the nearest institution and just take what is sent. Of course, one wishes that no institution sent out only partially trained persons, but unfortunately that cannot be said as yet; and, besides, many nurses work independently of any authority, and are sent by medical men and others who probably have no time to inquire into their qualifications. To this number, I fear, the "qualified nurse" will add a great many.

I have dwelt on this part of my subject because I feel so strongly that the establishment among us of this class of nurse—call her qualified, or assistant, or what you please—is a step, many steps, backwards; and because I desire to see Poor-law nursing lift up its head and reward those who have worked so long and so hopefully towards the better care of those unfortunate people who have come to our infirmaries.

(*To be continued.*)

### National Union of Women Workers.

### THE TRAINING OF MIDWIVES AND THE ORGANISATION OF THEIR WORK IN RURAL DISTRICTS.

The annual conference of the National Union of Women Workers was held this week at Cheltenham and Gloucester. The programme contained several items of interest to nurses, not the least so being the discussion on the afternoon of the first day on "The Training of Midwives and the Organisation of their Work in Rural Districts." Mrs. Creighton was in the chair, and the opening paper was read by Miss J. Wilson, member of the Midwives' Board, and president of the Midwives' Institute.

Starting from the point that the passing of the Midwives Act "imposes a duty on all those who realise the enormous importance to the nation of the health of its mothers and of the infants who will be its future workers," Miss Wilson spoke of the organisation necessary to make the Act a living force for good, giving her audience some useful facts respecting the powers of the local supervising authorities, the steps to be taken for notification and registration, etc. She touched upon the difficulty of the training question, no provision being made for meeting the heavy expenses that this necessary work will involve by any public body appointed under the Act, expressing the belief that such work, to be effectual, should be undertaken as a national duty to meet a national need. She advocated the formation of a National Council for training, the principal function of which would be to map out the country with local help for the organisation of training, the collection of funds, the founding of scholarships, and finally the establishment of a rational maternity hospital—doing, in fact, for midwives' work what the Queen's Institute does for nursing. In conclusion, Miss Wilson quoted the view of the author of "Mankind in the Making," that "our success or failure with this unending stream of babies is the measure of our civilisation."

Miss Alice Gregory followed with a very interesting paper, in which she pleaded for the more complete and thorough training of midwives under the new regulations, aiming steadily at a much higher standard of education than has yet been in force. Miss Gregory's own experience as a practising midwife in a country district led her to the belief that the profession should eventually be a self-supporting one. The indifference of the public to the whole question in the past had led to the present state of things, and it was a national duty to pull the profession out of the mire into which it has fallen; to prove to educated women that it was a possible means of earning a livelihood; and to provide them with a training school where at slight expense they might qualify for the great battle before them.

Mrs. Martin and Miss Amy Hughes opened the discussion, the latter speaking from her great experience as superintendent of the County Nursing Associations under the Queen's Jubilee Institute of the good work done by the village women, trained as midwives, but pointing out the immense importance of such women being under constant and fully trained supervision.

Mrs. Rickman, of the Rural Midwives' Association, made a somewhat astonishing speech, in which she deprecated educated and fully trained midwives taking "the bread out of the mouths" of the class of women from amongst whom she considered midwives should be chosen—*i.e.* "the superior artisan class."

Amongst other speakers were Colonel Griffiths, Chairman of the Registration Committee of the Gloucestershire County Council, who mentioned that the Council intended to keep in their own hands the administration of the Act, and asked for enlightenment on several points; Miss Lucy Robinson, representing the Association for Promoting the Training and Supply of Midwives, who gave a brief explanation of the objects of that Association, the most important being the establishment of a Central Bureau of Information on all subjects connected with the work of midwives; and Mrs. Percy Boulnois, who urged the formation of "loan funds" to solve the financial difficulty of training a sufficient number of midwives to meet the demand.

### Appointments.

Birkenhead and Wirral Children's Hospital.—Miss J. A. Chapman has been appointed Lady Superintendent. She was trained at the Western Infirmary, Glasgow, and has since been charge nurse at the Fountain Fever Hospital, Tooting, and holiday sister, ward sister, and assistant matron at the Royal Hospital for Sick Children, Edinburgh.

City Hospital, Park Hill, Liverpool.—Miss M. Christie has been appointed assistant matron. She was trained at Toxteth Infirmary, Liverpool, and has since been charge nurse and night superintendent of Prescot Infirmary, and night superintendent at the City Hospital, Park Hill, Liverpool.

Epsom Cottage Hospital.—Miss Florence Nightingale has been appointed staff nurse. She was trained at St. George's Infirmary, Fulham Road, London.

Herefordshire General Hospital.—Miss Marian Measures has been appointed matron. She was trained at Guy's Hospital, London, and her subsequent appointments have included sister-in-charge of the Nurses' Home, Bristol Royal Infirmary, sister and night sister of the Manchester Children's Hospital, assistant matron of the Seaman's Hospital, Greenwich, and matron of the Gravesend Hospital. She has on two occasions taken holiday duty at the Herefordshire Hospital.

Kilsyth Fever Hospital.—Miss Mary Chalmers has been appointed matron. She was trained at Helensburgh Fever Hospital.

King's Lynn and West Norfolk Hospital.—Miss Helen Swain has been appointed matron. She was trained at the Royal Berkshire Hospital, Reading, and has since been sister and night superintendent at the General Infirmary, Worcester. She also served in South Africa for two years and a half as a member of the Army Nursing Service Reserve.

Lambeth Infirmary, Brook Street, Kennington Road, London, S.E.—Miss F. M. Roberts-Thompson has been appointed night superintendent. She was trained at the Middlesex Hospital, where she afterwards became ward sister and night superintendent. She was trained in midwifery at Queen Charlotte's Hospital, London.

Montagu Cottage Hospital, Menborough.—Miss Annie Berry has been appointed working matron. She was trained at the Royal Infirmary, Derby, and has since been engaged as nurse at Chester, Retford, and Loughborough.

Poplar and Stepney Sick Asylum (Blackwall Branch).—Miss Mabel Ethel Maughan has been appointed superintendent nurse. She was trained at Mile End Infirmary where she has since been staff nurse and ward sister. She has also been doing private nursing at Bournemouth.

St. Mary's (Islington) Infirmary, Highgate Hill, London.—Miss S. A. Cook has been appointed sister. She was trained at Chelsea Infirmary and has since been staff nurse at St. Mary's (Islington) Infirmary, Highgate Hill, N.

Sudbury Infirmary.—Miss Elizabeth Pendrey has been appointed superintendent nurse. She was trained at Reading Union Infirmary in conjunction with Berks Hospital, and has since been charge nurse at Reading Union Infirmary, and district nurse at Twyford.

Tonbridge Union Infirmary, Pembury.—Miss R. M. Jones has been appointed charge nurse. She was trained at the Tonbridge Union Infirmary.

Woolwich Union Infirmary.—Miss Janie Louise Woodworth has been appointed ward sister. She was trained at Salford Union Infirmary. She has since been charge nurse at Dewsbury Union Infirmary.

Yarrow-on-Tyne Memorial Hospital.—Miss Alice Robson has been appointed matron. She was trained at the North Riding Infirmary, Middlesbrough-on-Tees, where she was afterwards sister in charge of one of the male wards.

### Presentations.

Salterhebble Hospital.—Miss Smithers, the lady night superintendent of the Salterhebble Hospital was presented on the 16th of last month with a gold enamelled pendant watch, a pearl enamelled brooch, and an autograph album, mounted in silver, containing the names of the 36 people attached to the hospital who subscribed to the present.

County Asylum, Rainhill, near Liverpool.—Miss H. M. Crerar, who for the last three years has been assistant matron at the County Asylum, Rainhill, on leaving to take up her duties as matron of the Royal District Asylum, Dundee, was presented by the officers and nursing staff with a handsome case of silver toilet requisites as a token of their esteem and good wishes.

West Ham and East London Hospital.—Miss Florence Glanville, in view of her approaching marriage having resigned the position of night sister of the West Ham Hospital, which she has held for three-and-a-half years, has been presented by the medical staff and committee with a handsome half-hoop diamond ring, accompanied by an illuminated testimonial. The nursing staff, past and present, together with the house surgeons and secretary, gave her a handsome case of cutlery, and the domestic staff a silver fish slice and fork, bread-brush and crumb-tray, and embroidered mats.

### Everybody's Opinion.

### THE ANGLO-AMERICAN NURSING HOME AT ROME.

"A Resident in Rome" writes under date October 29th: In your issue of October 17th "A Nurse," writing from the Anglo-American Home in Rome, states that she and five others have returned. As a resident in Rome I happen to know that this is not true. A free-bed case was refused last week because there was only one nurse in the home at the time. Only two nurses have as yet come to Rome, one of whom was nursing a case outside."

### MIDDLE-AGED NURSES.

"Nurse B." writes: I read with much interest the letter with the above heading, and I entreat my sisters of 40 and upwards not to lose heart but rather to thank God for all the years of active service they have been able to render. If in some ways our younger sisters should be accounted more up-to-date, we need never feel out of the running because our age and greater experience should at least have taught us many graces which the younger ones have still to learn, such as more patience, tact, and a quiet glad contentment. These can only come when we have reached middle age. We do not all attain the summit of our ambition, but to live each day and feel we have done some blessed work that in our young days we might have thought very uninteresting which will, if performed with a single eye, bring to us a rich harvest of gain. As regards the financial point; for my own part, I do not care so long as I am not obliged to be dependent upon those I love. Happiness does not consist in the abundance of things one has, and if one is blest with health and strength, a good nurse, be she quite old, will always find there is something for her to do.

### FRAUDS AND NURSING HOMES.

"E. C. H." writes: Matrons of nursing homes should, I think, be warned against a lady dressed in black who called on Sunday, October 18th, at a home in a suburb and engaged a room for a patient on the following morning. She named well-known doctors, and said that the case (a boy who had met with an accident at a public school) was to come in an ambulance, and be operated on the next day to his arrival. She entered into minute details and arranged fees. She said she was an Army nurse herself and had been trained at Netley. The boy did not arrive, and the master of the school was written to and said the whole story was a fiction. The lady was never left alone when she called at the home, so probably her motive for coming was frustrated. This may be the same person who was mentioned in your columns last week, and her visits ought to be put a stop to.

### POOR LAW VACANCIES AND GENERAL HOSPITAL-TRAINED NURSES.

"Margaret" writes: I am a workhouse-infirmary-trained nurse, and I want to ask if it is right that all the best Poor Law vacancies should be filled by nurses trained in general hospitals. In the Nursing Section of this week's Hospital, under "Appointments," I see that all the workhouse infirmary appointments have been obtained by nurses trained in general hospitals. Probably hundreds of well-qualified infirmary-trained nurses had applied for those posts, only to be put aside in favour of outsiders. This is an injustice to us and a slight to Poor Law training. If general hospital appointments were open to us, of course things would be equal, but they are not; general hospitals will not have us. What are we to expect when we have our three years' certificate and wish for a responsible post? Are we to remain probationer-nurses, or, if we are lucky, sisters, for ever? The prospect is not fascinating to a nurse with any ambition and is not likely to encourage intelligent women to enter the Poor Law service. Let hospital-trained nurses keep to hospitals unless hospitals and infirmaries will co-operate—which might be to the advantage of both. Will not the Workhouse Nursing Association, or someone with influence, take up our cause and represent it to the Local Government Board? I am sure that all workhouse nurses would be grateful to them if they would. I hope that your columns will be open to further correspondence on this subject and that Poor Law nurses will take it up.

### Echoes from the Outside World.

### The King at the Middle Temple.

MONDAY was Grand Night of Michaelmas term, and the King as a Bencher of the Inn dined in hall at the Middle Temple. There had been about 800 applications for the 200 odd seats, consequently ballot was resorted to, with the result that there were many disappointments. To suit the convenience of the Royal visitor, the hour for dinner was arranged two hours later than usual, and it was nearly 8 o'clock when the head porter, according to custom, knocking loudly on the door with his long ebony staff to gain admission, entered the right-hand door of the beautiful hall followed by the procession of Benchers and guests Later the King appeared, and the company assembled looked as if they wished to cheer, but that would have been a breach of time-honoured tradition, and his Majesty was allowed to walk up the hall in solemn silence. The tables were decorated with red and white flowers and tall candles for ornament, not use, as the light is supplied by electricity. After dinner the toast of "The King" was given, the House joining in singing the National Anthem; "The Queen," his Majesty himself setting the example in rising in response to it; "Domus" and "Absent Members." After the loving-cup had been passed round, the King lighted a cigar, a precedent which he had set in 1887. As a rule no smoking is allowed in hall.

### The Queen and Workmen's Dwellings.

THE suggestion of the Queen when she visited the workmen's dwellings erected by the London County Council on the Millbank Estate, that the provision of more cupboard space would be a boon to the tenants, has borne fruit. The Housing of the Working Classes Committee now report that, recognising the usefulness of her Majesty's suggestion, they decided to begin by having cupboards constructed in the tenements in Adelaide, Sydney, and Melbourne buildings which had previously been erected at Poplar. This work has now been completed, and the committee are taking steps to ensure the provision of good cupboard space in all the dwellings under the control of the Council.

### Death of Countess Spencer.

THE last time the Countess Spencer appeared in public was on the occasion of King Edward's Coronation in Westminster Abbey, and on Saturday afternoon her long illness terminated in death. For nearly fifty years Lady Spencer was one of the leaders of Society. She was honoured with the friendship of Queen Alexandra, who a few days ago sent a personal letter of inquiry as to her health, and the news of her death was at once telegraphed to the King and Queen. Born in 1835, Miss Charlotte Seymour, who was the granddaughter of Admiral Lord Hugh Seymour, married Lord Spencer in 1858. In Ireland, where her husband was Viceroy from 1868 to 1874, and again from 1881 to 1885, she is still remembered for the dignity and charm with which she fulfilled the social and ceremonial duties of her position. In London for years she made Spencer House the scene of gracious hospitality, and messages of condolence have been received from all quarters by Lord Spencer.

### Fire at the Vatican.

ON Sunday evening a fire broke out at the Vatican and except for the strenuous efforts of the Papal firemen and the Italian brigades, which at the instance of the Pope were hurriedly telegraphed for, would have been much more disastrous in its results. The first alarm was given at a quarter past eight, when smoke was seen issuing from the apartment occupied by M. Marle, the well-known restorer of ancient books. The gendarmes broke open the doors and found M. Marle in a heavy sleep. It is supposed that a fire in the kitchen had been forgotten, and that it had ignited some ammable material close to it. Unfortunately the furniture in the room was very old and the roof was of wood, which much increased the difficulties of the firemen. However, by a quarter past eleven the worst was over, though it was one o'clock before all was considered safe. Many treasures were rescued, but some were injured by water, notably the valuable private library of Leo XIII. which Father Ehrle had been rearranging. The Pope was kneeling in the chapel at prayer when the fire occurred. He immediately went to the scene of the conflagration, and gave orders as to what measures were to be taken.

### A Great German Scholar.

THE death of Dr. Theodor Mommsen, at the age of 86, has deprived the world of letters, as well as Germany, of one of its greatest historians, its greatest scholar. All his life through he had worked incessantly, "without hurry but without pause," and increasing years brought apparently no failure of mental power. He had been writing and studying as assiduously as usual up to a couple of days before his death, but it is thought that his constant attendance upon his wife, who has for some time been seriously ill with dropsy, had tried him more than he was aware. A stroke of paralysis suddenly stopped his work, and he passed peacefully away. Professor Mommsen was the son of a pastor, and came of a gifted family. The first volume of his Roman history—the publication of which did so much to make him famous—appeared in 1854. Many of his other works dealt with ancient Rome, and relate to linguistic and monetary questions and to inscriptions. He was very absent-minded, and it is said that when his first baby cried he picked it up and put it into the waste-paper basket. Another time he noticed a little girl in the road looking at him intently, and stopped and asked her who she was, being surprised to learn that she was one of his own twelve children!

### Lady Stewards.

THE Hospital and Home for Incurable Children, Maida Vale, has inaugurated a novel departure in the giving of charity dinners which, if it proves a success, will probably become very popular. Hitherto at a public dinner given at an hotel or at some well-known rooms, though occasionally ladies have been allowed to attend, they have never taken any very important part. Now on the 25th instant at Prince's Restaurant they will have an opportunity of showing their capacity to act as stewards. The list is a long one, including the Duchess of Marlborough, the Marchioness of Granby, the Countess Annesley; and it has been arranged that ladies bringing parties shall have tables reserved for them, thus making the function both more exclusive and more attractive than an ordinary public banquet. The Duke of Connaught has promised to preside, and it is expected that a handsome sum will be available to hand over to the treasurer of the Hospital.

### Mr. Whistler's Pictures.

Messrs. Obach have collected together about 300 of Mr. Whistler's pictures and are now exhibiting them at their Gallery in New Bond Street. The collection includes some of the best etchings of the wonderful artist, and those who do not know his works intimately will be glad to see them. The "Thames Set" is there, of which the most typical is "The Pool," also other examples of scenes on the same river, of which "The Troubled Thames" and "Price's Candle Works" should be specially noticed. There are early examples of his art, as in the French set, and a beautiful Venetian series. None should be missed, almost every picture—and most of them are very small—are gems of art. The walls of the gallery are hung with yellow silk for the occasion, and save that it is a little full in tint, the innovation contributes to the success of the show.

### A Book and its Story.

### A BOOK OF THE YEAR.*

"Treasure and Heart" is a very charming book. Written with refinement, vivacity, and intense insight, it holds the reader, not only by these qualities, but by a certain sincerity in the writer, which suggests that its production has been a labour of love, carefully conceived and faithfully carried out. It will undoubtedly take its place as one of the books of the year.

The plot has no claim to originality. It hangs on the discovery of mislaid letters, and other clues, necessary to establish the identity of the heroine, Cara Lassburgh. The reader follows her through the mazes of a thorny way with unabated interest. She makes her first appearance as a tiny child at an auction of rare curios in Havre. There she had been taken by a woman on the look out for bargains. Finding herself unwatched, with all the confidence of feminine babyhood, she had slipped off the knee of Mdme. Leblanc and had proceeded down a table spread with treasures in china, and quietly annexed a little black figure of Africa that she had been eyeing for some time. Possessed of this, and unheeding the cries of Mdme. Leblanc, she plodded on valiantly, until, with a headlong rush, she fell into the arms of Gian Lorenzo. There, with her head against his grizzled beard, she peeped roguishly round as if at some dreaded object. "A strange thrill penetrated Lorenzo's heart—the empty heart which was made for love, and held only a dead image. He held the little girl with fatherly tenderness, and his expressive eyes flashed and flamed defiance." This sudden change of expression was excited by the threats of the enraged woman from whose unwelcome custody little Cara had freed herself when she made for the welcome haven of Lorenzo's arms. "The child clung to his neck, and he felt that he would sooner have parted with the Gubbio lustre and a year's profits than give her up to the virago with the threatening eye." He found, upon inquiry, that Cara was a little English girl whose father had fallen ill in the house of Madame Leblanc. There he had rented rooms. But a sudden end had come to his illness, and little Cara, or "Callie," as she called herself, was parentless. She had not yet learnt this when "she pattered into Gian Lorenzo's empty heart, as she had pattered, among porcelain and pottery, into his kind arms." In glancing round the barely-furnished rooms Lorenzo's attention had been arrested by a large canvas standing alone, without a frame. With the eye of a connoisseur he perceived that it was a painting of considerable value. Nearer inspection proved it to be the portrait of a lady—"the lady with the pearls"—a fine specimen of the English school, of Sir Joshua, Romney, perhaps a Gainsborough. Lorenzo, after inspecting it carefully, withheld any expression of satisfaction which the discovery had aroused. Leblanc and his wife knew nothing of art nor the value of the treasure which was the property of the dead Englishman. There was also a seal ring which Lorenzo bought. After some bartering, he replied, in response to a suggestion of Madame Leblanc's, "that to a wealthy amateur the price asked was a mere bagatelle, 'I am no amateur with a fortune to spend.' I am a dealer, too, but I will give you six hundred francs for the picture and the child together." So the bargain was made, and Cara passed from the hands of the Leblancs into the safe keeping of her self-selected guardian. "As for the dead stranger, Lorenzo undertook to pay the costs of the funeral and the little girl was delivered from thraldom, no more smacks and no more scowls." Cara was perfectly happy in her new domicile. Lorenzo introduced her as his grandchild, and confided her to the loving care of his faithful servant, Teresina. Teresina, with others, did not believe in the relationship, "but considered it an affront in anyone else to doubt what her master chose to assert." Lorenzo lived in fear of English relatives appearing, but as the English Vice-Consul at Havre, had only made a hasty note about the child, as he was on the point of leaving for the Mediterranean, when Lorenzo had called upon him before bringing her to Florence, he hoped they would not be able to trace her by this means. Cara, brought up in the old-world atmosphere of the antiquary's shop, found great delight in its curious collection of treasures. "Its street front was extremely modest, but the whole premises went like a mole's run in and out of the backs of houses along the street, sometimes mounting upstairs, sometimes going down into cellars. Its treasures were well known to Florentines and to foreigners, whose annual spending contributes so largely to Italy's revenue, but to whom Italy gives better than she receives." Here was Cara's playground. The following passage expresses intelligibly the subtle sensations which a repository like Lorenzo's excite in a reflective mind:—"Each soul entering the world brings with it its own mystery and imparts something of it to the thing it touches—things made by human hands—not machinery, which have shared a short human life. So a subtle magic haunts the place of such flotsam and jetsam, and the possessions of the dead have a teaching of their own unknown to science."

Cara's happy childhood passed dreamily away and at fourteen she was lovely. Graceful and unconscious she moved about in the shadowy old house, lighting up its dark corners with her bright presence, varying her pleasures by feeding the pigeons at the corner between Uffizi Palace and the Loggia dei Lanzi and playing with the young Marchese di Sotelli. For music she had an unusual gift, and a most quaint scene is presented when the young Marchese wandering in the mazes of Lorenzo's stores comes upon the child musician, playing a solo of consolation to "The Lady of the Tears." The picture was as real to her as a living being. It had shared her childish confidences ever since she could remember. This is the peep we get of her before the picture which plays an important part in her story. It is Marcomio's first sight of Cara. He found it difficult to be quite sure if she was "real," so absorbed and wrapt was she in her attitude of inspiration and devotion. He had been drawn into the little room by the sound of Cara's viola. On a large easel, facing the intruder, stood the picture of "the lady with the pearls." "There were ropes of pearls about her neck, and unshed tears in her eyes. She was playing on an old spinet. The beautiful face, in a cloud of dark hair twisted with pearls, was tilted slightly upwards." Cara, in a carved oak chair, was drawn up in front of the picture, her cropped head of dark hair showing over the back of it. She was busily accompanying the lady with the spinet on her viola; a handsome tabby cat on her lap, his smug face tied in a scarlet kerchief, making spasmodic interruptions to the music.

There are many other characters that are distinctive and lifelike, including Colin—Cara's lover—Treflée, the friend of Marcomio's Irish stepmother, and others. As for the stepmother herself, Lady Susan Fitzgarratt, with her passion for "collections, her warm heart, and her purring voice,' she is intensely diverting. In appearance she is described as "a comfortable bundle of old clothes," but she is also a delightful bundle of generous inconsistencies.

* "Treasure and Heart." By Mary Deane. (John Murray. 6s.)

### For Reading to the Sick.

"I WILL GIVE YOU REST."

"Come unto Me, ye weary,
And I will give you rest."
O blessed voice of Jesus,
Which comes to hearts oppresst;
It tells of benediction,
Of pardon, grace, and peace,
Of joy that hath no ending,
Of love which cannot cease.

"And whosoever cometh,
I will not cast him out."
O welcome voice of Jesus,
Which drives away our doubt;
Which calls us very sinners,
Unworthy though we be,
Of love so free and boundless,
To come, dear Lord, to Thee.

*W. C. Dix.*

The Christian may have, must have, an outer life in the world, of training, toning, educating—in fact, of "tribulation"; but with equal certainty he has a true life, an inner life, "in Christ."

The character of the inner life—as of the majestic life of the Eternal even in His Passion—is this, "in Me ye may have peace."

"In the world ye shall have tribulation" is a necessary teaching of the Cross. But then—such is the illuminating revelation of the Resurrection under the interpreting energy of the Spirit of God—the "tribulation" is turned to excellent uses. Trial is the school of obedience; trial is the means of the growth of character; trial is the method of discipline; trial is the training of faith. There is this sad fact of the outer life of the Christian; but the silence of the winter world witnesses to the coming life of spring; the narrow wrapping of the narrow bud witnesses to the opening flower; the dark night witnesses to the morning; the outer struggle of the Christian witnesses to the Inner Life.

Let righteousness be your study, and strength your duty, and love which purifies, the very atmosphere of your being. Avoid that which is evil, cleave to that which is good; and there lies before you a future of unimagined wonder and unmeasured blessedness. Beloved, let us live for the ideal; and then "it doth not yet appear what we shall be, but we know that we shall be like Him, for we shall see Him as He is."—*Canon Knox Little.*

If I find Him, if I follow,
What His guerdon here?
"Many a sorrow, many a labour,
Many a tear."

If I still hold closely to Him,
What hath He at last?
"Sorrow vanquish'd, labour ended,
Jordan past."

If I ask Him to receive me,
Will He say me nay?
"Not till earth, and not till Heaven
Pass away."

*T. M. Neale, D.D.*

### Notes and Queries.

FOR REGULATIONS SEE PAGE 21.

*Light Treatment.*

(48) Have any cases been known of a simple adenoid growth in the rectum, for which colotomy has been performed with a view to cure, being benefited by light treatment or electricity?—*L.A.M.*, Wales.

It is quite possible that such cases have been known, but we have not heard of one.

*Convulsions.*

(49) Will you kindly tell me if acute bronchitis and convulsions are ever brought on by teething in a child 10 months old?—*E. K.*

A case like this should be attended by a medical man, who will be able to answer this question.

*Manchester Hospitals.*

(50) You will oblige me very much by letting me know which hospitals in Manchester are connected with the Dublin hospitals? —*M. O'B.*

There is no connection between the hospitals of the two cities.

*Labourer's Children.*

(51) Is there any home where a labourer could place his motherless children? The mother has just died of rapid consumption, leaving a baby a month old.—*Nurse G.*

Apply to the Local Government Board, Whitehall, London, S.W., for particulars, which are supplied gratis, as to boarding-out children.

*Advice.*

(52) 1. I should be very much obliged by any suggestions as to any means, other than by advertising, by which I might make known my wish to receive patients. My home is very suitable for those requiring the open-air treatment. 2. Can you suggest any employment for a fully-trained nurse who has become partially deaf?—*Sister.*

1. If you object to advertising, your only alternative is to ask your friends to recommend your home. 2. Why not try massage?

*Midwifery.*

(53) Can you kindly tell me if a trained nurse (three years) holding the L.O.S. and the certificate for midwifery from the Hospital for Women, Brighton, is, or is not, a midwife under the new Act?—*R. U. D.*

Certainly.

*Jamaica.*

(54) I should be glad if you can tell me how to obtain a berth in Jamaica, and what chance a trained nurse has of getting an appointment in a hospital there.—*Nurse L.*

Apply to the Matron, the Public Hospital, Jamaica.

*Lady Dufferin's Fund.*

(55) *Nurse B.* would be very pleased to have particulars of Lady Dufferin's Fund, or any well-known private nursing home for paying patients in India.

The Matron of the Lady Dufferin Victoria Hospital, Calcutta, will give you any information about the fund. We do not recommend private nursing homes.

*Locality.*

(56) My brother is a masseur, and I am a masseuse as well as a trained nurse. We wish to take a small home and receive patients, and we would be glad if you could recommend us a suitable locality in which to start.—*A. S. B. M.*

We never give advice on such subjects; but if you know any specialist on nervous diseases who would be likely to send you patients, you might consult him about it.

**Standard Nursing Manuals.**

"The Nursing Profession: How and Where to Train." 2s. net; 2s. 4d. post free.

"Nursing: Its Theory and Practice." (Revised Edition). 3s. 6d. post free.

"Elementary Anatomy and Surgery for Nurses." By William McAdam Eccles, M.D.Lond., M.B. 2s. 6d. post free.

"Elementary Physiology for Nurses." By C. F. Marshall, M.D., B.Sc., F.R.C.S. 2s. post free.

"Ophthalmic Nursing." By Sydney Stephenson, M.B., F.R.C.S., 3s. 6d. net; 3s. 10d. post free.

"Nursing in Diseases of the Throat, Nose, and Ear." By P. Macleod Yearsley, F.R.C.S.Eng., M.R.C.S. 2s. 6d. post free.

"Fevers and Infectious Diseases." 1s. post free.

## Figures and Tables

**Figs. 1 and 2. f1:**
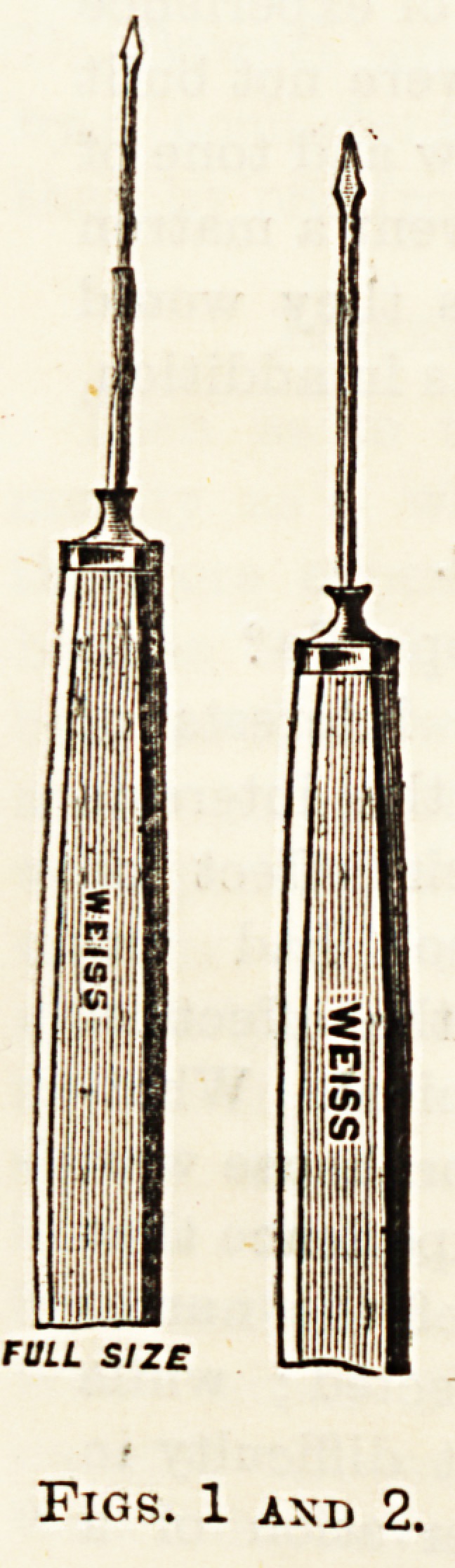


**Fig. 3. f2:**
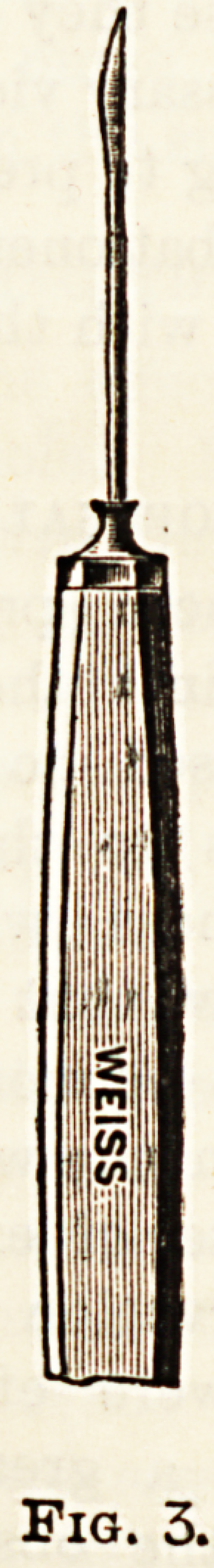


**Fig. 4. f3:**
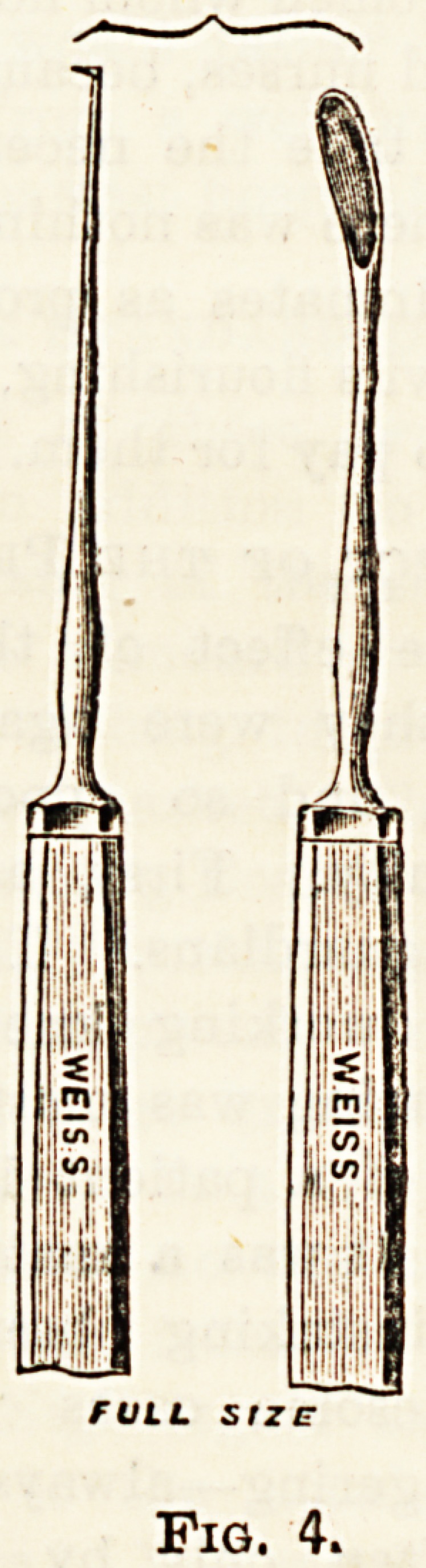


**Fig. 5. f4:**
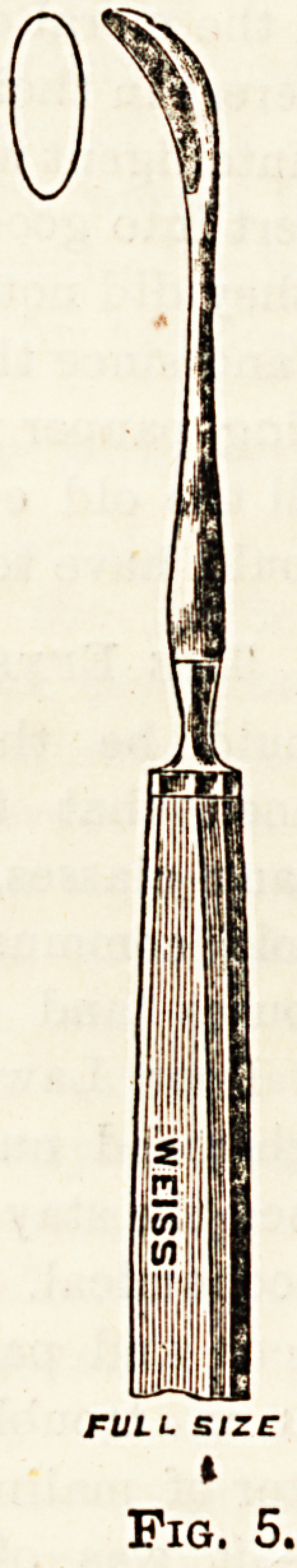


**Fig. 6. f5:**